# The Rametrix^™^ PRO Toolbox v1.0 for MATLAB^®^

**DOI:** 10.7717/peerj.8179

**Published:** 2020-01-06

**Authors:** Ryan S. Senger, John L. Robertson

**Affiliations:** 1 Department of Biological Systems Engineering, Virginia Polytechnic Institute and State University (Virginia Tech), Blacksburg, VA, United States of America; 2 Department of Chemical Engineering, Virginia Polytechnic Institute and State University (Virginia Tech), Blacksburg, VA, United States of America; 3 DialySensors, Inc., Blacksburg, VA, United States of America; 4 Department of Biomedical Engineering and Mechanics, Virginia Polytechnic Institute and State University (Virginia Tech), Blacksburg, VA, United States of America; 5 Carilion School of Medicine and Research Institute, Virginia Polytechnic Institute and State University (Virginia Tech), Blacksburg, VA, United States of America

**Keywords:** Raman spectroscopy, MATLAB, Principal component analysis, Discriminant analysis, Spectral data analysis, Prediction, Urine, Nephrology

## Abstract

**Background:**

Existing tools for chemometric analysis of vibrational spectroscopy data have enabled characterization of materials and biologicals by their broad molecular composition. The Rametrix^™^ LITE Toolbox v1.0 for MATLAB^®^ is one such tool available publicly. It applies discriminant analysis of principal components (DAPC) to spectral data to classify spectra into user-defined groups. However, additional functionality is needed to better evaluate the predictive capabilities of these models when “unknown” samples are introduced. Here, the Rametrix^™^ PRO Toolbox v1.0 is introduced to provide this capability.

**Methods:**

The Rametrix^™^ PRO Toolbox v1.0 was constructed for MATLAB^®^ and works with the Rametrix^™^ LITE Toolbox v1.0. It performs leave-one-out analysis of chemometric DAPC models and reports predictive capabilities in terms of accuracy, sensitivity (true-positives), and specificity (true-negatives). Rametrix^™^PRO is available publicly through GitHub under license agreement at: https://github.com/SengerLab/RametrixPROToolbox. Rametrix^™^ PRO was used to validate Rametrix^™^ LITE models used to detect chronic kidney disease (CKD) in spectra of urine obtained by Raman spectroscopy. The dataset included Raman spectra of urine from 20 healthy individuals and 31 patients undergoing peritoneal dialysis treatment for CKD.

**Results:**

The number of spectral principal components (PCs) used in building the DAPC model impacted the model accuracy, sensitivity, and specificity in leave-one-out analyses. For the dataset in this study, using 35 PCs in the DAPC model resulted in 100% accuracy, sensitivity, and specificity in classifying an unknown Raman spectrum of urine as belonging to a CKD patient or a healthy volunteer. Models built with fewer or greater number of PCs showed inferior performance, which demonstrated the value of Rametrix^™^ PRO in evaluating chemometric models constructed with Rametrix^™^ LITE.

## Introduction

Through advances in instrumentation, vibrational spectroscopy, including Raman spectroscopy, has become rapid, portable, and inexpensive (Zarei, 2017; Crocombe, 2018), making it ideal for use in screening assays of biological fluids, cells, or other materials.  The spectrum obtained by Raman spectroscopy is representative of the molecular composition of that sample but can be complex and difficult to deconvolute into its molecular components.  This is especially true of biological samples (Movasaghi, Rehman & Rehman, 2007; Athamneh et al., 2014; Zu et al., 2015; Butler et al., 2016).  The differentiation between healthy and urine from patients with chronic kidney disease (CKD) was done by a chemometric analysis of Raman spectra using principal component analysis (PCA) and discriminant analysis of principal components (DAPC) (Fisher et al., 2018).  In a chemometric analysis, Raman spectra are treated as “fingerprints”, and multivariate statistical tools discover unique features and similarities among spectra (Shinzawa et al., 2009; Gautam et al., 2015).  Software packages such as CytoSpec^™^ (http://www.cytospec.com), Unscrambler^™^ (https://www.camo.com/unscrambler/), and others include such tools.  The Raman Chemometrics (Rametrix^™^) LITE Toolbox (Fisher et al., 2018) was created for MATLAB^®^ to further streamline the creation of Raman-based chemometric screens.  It offers tools for Raman spectral processing along with PCA, DAPC, and other tools for spectral comparisons in an easy-to-use graphical interface.  It is also offered publicly through GitHub.

Here, the companion Rametrix^™^ PRO Toolbox v1.0 for MATLAB^®^ is introduced.  It is also offered publicly through GitHub and provides additional functionality to the Rametrix^™^ LITE Toolbox.  In particular, it evaluates DAPC models using a leave-one-out procedure.  Metrics are reported regarding the prediction accuracy of the model, including sensitivity (true-positive rate) and specificity (true-negative rate).   Rametrix^™^ PRO was used to evaluate the chemometric DAPC models published in Fisher et al. (2018) that classify Raman spectra of urine as belonging to healthy individuals (i.e., “healthy”) or CKD patients (i.e., “unhealthy”).

## Materials & Methods

### Software and calculations

Raman spectra files (in .SPC format) were processed using the Rametrix™ LITE Toolbox in MATLAB r2018a (MathWorks; Natick, MA) as described previously (Fisher et al., 2018).  Briefly, spectra were (i) truncated to include a Raman shift range of 400–1,800 cm^−1^, (ii) baselined using the Goldindec algorithm (Liu et al., 2015) (baseline polynomial order = 3; estimated peak ratio = 0.5; smoothing window size = 5), (iii) vector normalized, and (iv) scan replicates averaged for each patient.  PCA and DAPC models were also built using the Rametrix^™^ LITE Toolbox.  Multiple DAPC models were produced by varying the number of principal components (PCs) used in model construction.

The Rametrix^™^ PRO Toolbox v1.0 was used to perform leave-one-out analysis on all DAPC models.  Spectra classification for each left-out spectrum (i.e., “healthy” or “unhealthy”) was predicted and compared to the actual classification.  The averaged spectrum from each healthy individual or CKD patient was excluded from model construction and predicted in the leave-one-out routine.  Thus, the leave-one-out validation was done with respect to individual specimens and individuals, not according to scan replicates.  Model accuracy was calculated as the percentage of spectra where classification was predicted correctly.  Sensitivity (i.e., the true-positive rate) and specificity (i.e., the true-negative rate) were also calculated and reported as percentages.

Rametrix^™^ PRO also has the capability to calculate “random chance” values of prediction accuracy, sensitivity, and specificity for any dataset.  While this may be obvious for datasets with only two possible classifications (i.e., “healthy” or “unhealthy”), it is less obvious for datasets with multiple potential classifications with unequal representation.  In these cases, the calculated accuracy, sensitivity, and specificity of leave-one-out validation routines are best presented relative to their random chance values. 

### Public access

The Rametrix^™^ PRO Toolbox v1.0 is available through GitHub with an MIT license agreement (https://github.com/SengerLab/RametrixPROToolbox). The dataset of raw Raman spectra files used in the analysis here is also available for download with the Rametrix^™^ PRO Toolbox on GitHub.  In addition, the Rametrix^™^ LITE Toolbox v1.0 and v1.1 is also available through GitHub (https://github.com/SengerLab/RametrixLITEToolbox) under license agreement (Fisher et al., 2018).

### Specimens collected

Voided urine specimens were collected from 20 healthy human volunteers on the Virginia Tech campus and 31 patients with CKD Stage 4–5, undergoing peritoneal dialysis therapy.  Single specimens were collected from the healthy human volunteers (*n* = 20), and multiple time-point specimens were obtained from the CKD patients (*n* = 118). Specimen storage and scanning details are published elsewhere (Fisher et al., 2018; Senger et al., 2019).  Briefly, specimens were stored at −35 °C prior to analysis. They were thawed and warmed to 37 °C and transferred to 1 mL glass vials (ThermoFisher Scientific; Waltham, MA) for analysis by Raman spectroscopy.

### Raman spectroscopy

Spectra were obtained using an Agiltron (Woburn, MA) PeakSeeker Raman spectrometer. Scans were obtained from liquid phase samples using a 785 nm laser operated at 100 mW with 10 s integration time.  A total of 10 scans were obtained and averaged for each sample.

### IRB approval

Informed written consent for the collection of urine specimens from healthy volunteers on Virginia Tech campus was obtained under protocol VT15-703 from the Virginia Tech Institutional Review Board.  Informed consent for the collection of urine specimens from patients with CKD was obtained under protocol RPP/177151.2 from Frenova (Fresenius Renal Research, Waltham, MA, USA). All urine specimens were de-identified and assigned a code upon collection. 

## Results

The leave-one-out analysis results, obtained with the Rametrix^™^ PRO Toolbox and the urinalysis dataset (i.e.,  *n* = 20 “healthy” and *n* = 118 “unhealthy” specimens), are given in Table 1. The analysis was repeated for DAPC models built using different number of PCs. Typically, as the number of PCs is increased in DAPC model-building, the separation of clusters improves. However, we find this can be misleading. As the number of PCs used to build the DAPC model surpassed 40 (which contained approximately 99.89% of the dataset variance), the prediction accuracy of the model decreased precipitously. For the urinalysis dataset analyzed in this study, DAPC models built with 35–38 PCs returned 100% accuracy, sensitivity, and specificity in leave-one-out analyses. Simpler models (i.e., built from 2–10 PCs) returned 99% accuracy, 100% sensitivity, and 95% specificity for identifying an unknown Raman spectrum of a urine specimen as coming from a “healthy” volunteer or “unhealthy” CKD patient. These far exceed the random chance accuracy, sensitivity, and specificity values of 50%. These results suggest that Raman spectroscopy and Rametrix^™^ technology can be used to classify effectively whether an unknown urine specimen is from a healthy individual or a CKD patient.

**Table 1:** “Leave-one-out” analysis of the urinalysis dataset using the Rametrix^™^ PRO Toolbox.

**Number of PCs in DAPC model**	**Dataset variance explained by PCs**	**Modelaccuracy**	**Modelsensitivity**	**Modelspecificity**
1	47.03%	97%	100%	80%
2	78.92%	99%	100%	95%
3	89.16%	99%	100%	95%
4	93.89%	99%	100%	95%
5	95.93%	99%	100%	95%
6	97.23%	99%	100%	95%
7	97.90%	99%	100%	95%
8	98.36%	99%	100%	95%
9	98.78%	99%	100%	90%
10	99.01%	99%	100%	90%
15	99.52%	99%	100%	90%
20	99.70%	98%	100%	85%
25	99.79%	99%	100%	95%
30	99.83%	99%	100%	95%
35	99.87%	100%	100%	100%
40	99.89%	99%	99%	100%
45	99.91%	95%	94%	100%
50	99.93%	92%	91%	100%
55	99.94%	83%	81%	100%
60	99.95%	72%	67%	100%
70	99.96%	29%	17%	100%
80	99.97%	22%	8%	100%
90	99.98%	14%	0%	100%
100	99.99%	14%	0%	100%

**Figure 1:**
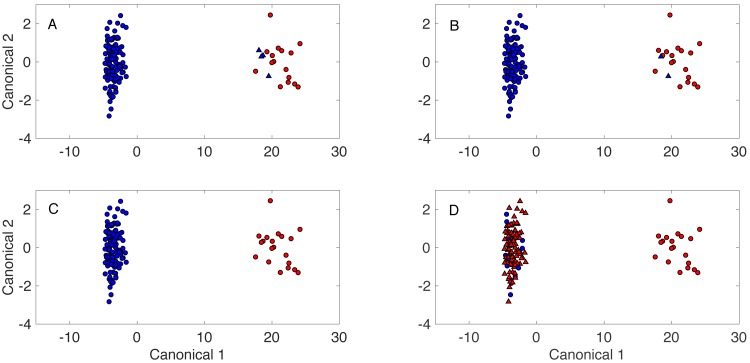
Spectral clustering by DAPC with predicted classifications. DAPC models were built with (A) 1 PC, (B) 10 PCs, (C) 35 PCs, (D) 70 PCs. Red denotes Raman spectra classified as healthy. Blue denotes Raman spectra classified as unhealthy. Data points appearing as a circle were classified correctly. Data points appearing as a triangle were classified incorrectly.

DAPC model clustering results are shown in Fig. 1 for models built with 2, 10, 35, and 70 PCs, respectively. PCA results, used to build the DAPC models, were presented previously with Rametrix^™^ LITE results (Fisher et al., 2018). In DAPC plots, each data point represents an entire Raman spectrum. Clustering is indicative of spectra recognized as similar for well-functioning models. In Fig. 1, those samples classified correctly are represented by circles, and those classified incorrectly are represented by triangles. Those classified as from healthy individuals are in red, and those from unhealthy CKD patients are in blue. The clustering between spectra of healthy and unhealthy specimens is apparent, as are the mis-classifications by DAPC models. This is readily apparent in the model built with 70 PCs (Fig. 1D), where likely model over-fitting resulted in several mis-classifications.

## Discussion

The Rametrix^™^ PRO Toolbox v1.0 comes with distinct functions that work with the Rametrix^™^ LITE Toolbox to validate DAPC spectra classification models. It generates a DAPC classification for an “unknown” sample and applies leave-one-out analysis over an entire spectral dataset. This process was used to evaluate DAPC models by blinding one sample to DAPC model building and treating it as an unknown. An additional function of Rametrix^™^ PRO generates random chance values of accuracy, sensitivity, and specificity, which can be used to put leave-one-out results into better perspective. For example, if accuracy, sensitivity, and specificity values are below expectations but exceed the random chance values, it is likely that more samples are needed to improve model performance. Thus, if random chance values are exceeded, the DAPC model is showing at least some success at classifying samples.

The leave-one-out procedure of Rametrix^™^ PRO was demonstrated using the urinalysis dataset described in our initial publication (Fisher et al., 2018) and described further here. Comparing the predicted and known classifications for all specimens allowed calculation of model accuracy, sensitivity, and specificity. Here, a “positive” result was the presence of CKD, and a “negative” result was the absence of CKD (i.e., healthy). A “true” result occurs when model prediction matches the known classification, and a “false” result occurs when these differ. Thus, the model accuracy is the percentage of true results (both positive and negative) over the entire dataset, where a value of 100% means that predictions for all specimens were correct. The model sensitivity (i.e., true-positive rate) gives the percentage of true results that were predicted correctly. This also means the percentage of urine specimens from CKD patients that were classified with the “unhealthy” label. The model specificity (i.e., true-negative rate) gives the percentage of false results that were predicted correctly. This value also represents the percentage of urine specimens from healthy volunteers classified as “healthy”.

While we admit the dataset size of this study is somewhat small (138 urine specimens), it suggests that Rametrix^™^ can be used as a screening technology to identify individuals with undiagnosed CKD. This study focuses on the analysis of urine specimens from CKD Stage 4–5 patients. Studies are underway that include a significantly larger number of patients and include CKD Stages 1–5. Identifying early-stage CKD is critical for nephrologists to prescribe treatments that can halt/slow CKD progression and save lives.

## Conclusions

The Rametrix^™^ PRO Toolbox v1.0, introduced here and available through GitHub with license agreement, enables leave-one-out evaluation of PCA and chemometric DAPC models produced by Rametrix^™^ LITE. It can also calculate the random chance accuracy, sensitivity, and specificity for any dataset. Raman spectroscopy is fast and inexpensive, and Rametrix^™^ ensures that Raman spectral signatures of the hundreds of molecular components are used in classifications. While the example in this manuscript focuses on classification of urine specimens, the Rametrix^™^ LITE and PRO Toolboxes can be applied to all studies involving chemometric data from Raman or other vibrational spectroscopy.

## Additional Information and Declarations

### Data Availability

The following information was supplied regarding data availability: The software and dataset are available at GitHub: https://github.com/SengerLab/RametrixPROToolbox.
